# Does a high dietary intake of resistant starch affect glycaemic control and alter the gut microbiome in women with gestational diabetes? A randomised control trial protocol

**DOI:** 10.1186/s12884-021-04366-4

**Published:** 2022-01-18

**Authors:** Cathy Latino, Emily J. Gianatti, Shailender Mehta, Johnny Lo, Amanda Devine, Claus Christophersen

**Affiliations:** 1 School of Medical & Health Sciences, Edith Cowan University, Joondalup, Western Australia Australia; 2 Institute for Nutrition Research, Edith Cowan University, Joondalup, Western Australia Australia; 3 Department of Dietetics, Fiona Stanley Hospital, South Metropolitan Health Service, 11 Robin Warren Drive, Murdoch, 6150 Western Australia Australia; 4 Department of Endocrinology, Fiona Stanley Hospital, Murdoch, Western Australia Australia; 5 Department of Neonatology, Fiona Stanley Hospital, Murdoch, Western Australia Australia; 6 Curtin Medical School, Curtin University, Bentley, Western Australia Australia; 7 School of Medicine, University of Notre Dame, Fremantle, Western Australia Australia; 8 School of Science, Edith Cowan University, Joondalup, Western Australia Australia; 9 Centre for Integrative Metabolomics and Computational Biology, Edith Cowan University, Joondalup, Western Australia Australia; 10 WA Human Microbiome Collaboration Centre - TrEnD Lab, School of Molecular & Life Sciences, Curtin University, Bentley, Western Australia Australia

**Keywords:** Gestational diabetes, Diet therapy, Resistant starch, Gut microbiome, Fasting glucose, Short-chain fatty acids

## Abstract

**Background:**

Gestational Diabetes Mellitus (GDM) is prevalent with lasting health implications for the mother and offspring. Medical nutrition therapy is the foundation of GDM management yet achieving optimal glycaemic control often requires treatment with medications, like insulin. New dietary strategies to improve GDM management and outcomes are required.

Gut dysbiosis is a feature of GDM pregnancies, therefore, dietary manipulation of the gut microbiota may offer a new avenue for management. Resistant starch is a fermentable dietary fibre known to alter the gut microbiota and enhance production of short-chain fatty acids. Evidence suggests that short-chain fatty acids improve glycaemia via multiple mechanisms, however, this has not been evaluated in GDM.

**Methods:**

An open-label, parallel-group design study will investigate whether a high dietary resistant starch intake or resistant starch supplement improves glycaemic control and changes the gut microbiome compared with standard dietary advice in women with newly diagnosed GDM. Ninety women will be randomised to one of three groups - standard dietary treatment for GDM (Control), a high resistant starch diet or a high resistant starch diet plus a 16 g resistant starch supplement. Measurements taken at Baseline (24 to 30-weeks’ gestation), Day 10 and Day 56 (approximately 36 weeks’ gestation) will include fasting plasma glucose levels, microbial composition and short-chain fatty acid concentrations in stool, 3-day dietary intake records and bowel symptoms questionnaires. One-week post-natal data collection will include microbial composition and short-chain fatty acid concentrations of maternal and neonatal stools, microbial composition of breastmilk, birthweight, maternal and neonatal outcomes. Mixed model analysis of variance will assess change in glycaemia and permutation-based multivariate analysis of variance will assess changes in microbial composition within and between intervention groups. Distance-based linear modelling will identify correlation between change in stool microbiota, short-chain fatty acids and measures of glycaemia.

**Discussion:**

To improve outcomes for GDM dyads, evaluation of a high dietary intake of resistant starch to improve glycaemia through the gut microbiome needs to be established. This will expand the dietary interventions available to manage GDM without medication.

**Trial registration:**

Australian New Zealand Clinical Trial Registry, ACTRN12620000968976p. Registered 28 September 2020

**Supplementary Information:**

The online version contains supplementary material available at 10.1186/s12884-021-04366-4.

## Background

Gestational Diabetes (GDM) is a state of glucose intolerance first discovered in pregnancy via routine screening undertaken between 24 and 28 weeks of gestation [1]. It is largely a disease of insulin resistance (IR) with the prevalence increasing in parallel with increasing rates of obesity [2]. Optimal glycaemic control reduces many of the risks associated with GDM [3–5] including preeclampsia, macrosomia, large for gestational age, shoulder dystocia and neonatal hypoglycaemia [2, 6]. Offspring of women with GDM also have a higher risk of obesity and impaired glucose metabolism [2, 7–10]. Mothers with a history of GDM have an increased risk of developing cardiovascular disease [11] and type 2 diabetes (T2DM) [12].

Current evidence-based dietary strategies are often insufficient to optimise glycaemia [13] therefore, in some populations, more than half of GDM women require pharmacotherapy to control their blood glucose [14–17]. This increases the burden to the woman and health system through additional monitoring and clinic appointments to ameliorate the risk [18]. Hence, new dietary strategies are required to improve outcomes for women and their offspring [5] and to reduce health expenditure [18].

Similar to T2DM [19, 20], gut dysbiosis has been reported as a feature of GDM pregnancies and associated with higher blood glucose levels [21–23]. Evidence to support the relationship between diet, the gut microbiota, IR and glycaemic control in T2DM is strengthening [24–30]. Hence, specific changes to the diet can modify the gut microbiota [31, 32] suggesting that dietary modifications which impact the maternal gut microbiota and metabolome are potential therapies to improve glycaemia in GDM [21, 33–36].

Gut microbiota and glycaemic control are known to be altered by fermentable dietary fibres such as resistant starch (RS) [28, 29, 37, 38]. A systematic review by Colantonio, Werner and Brown [29] concluded that foods with prebiotic properties, such as RS, may improve glycaemic control in women with T2DM. More specifically, a meta-analysis of RS supplementation by Wang et al. [28] showed improvements in fasting glucose and IR, particularly in overweight or obese people with diabetes. Microbial fermentation of RS increases the production of short chain fatty acids (SCFA) [39]. SCFA are thought to improve glycaemic control through multiple mechanisms [20], discussed later, suggesting that the manipulation of the microbiome using RS may be a novel therapeutic option for reducing the severity of GDM.

This study will evaluate whether a high dietary RS intake from diagnosis with GDM can improve maternal glycaemic control; impacts the maternal and/or neonatal gut microbiota, faecal SCFA production, maternal and neonatal health outcomes; and collect information to determine the health economic benefits of improvement of dyads health outcomes that result from this intervention.

We hypothesise that compared with standard GDM dietary advice, women with a high dietary intake of RS from the diagnosis of GDM will show a reduction in fasting blood glucose (FBG) levels and other measures of glycaemic control. This will reduce the percentage of women who require insulin and improve maternal and foetal outcomes.

## Methods

### Overview of study design

An open-label, parallel-group design study will be used to investigate whether a higher RS intake from diagnosis of GDM changes the gut microbiome and improves glycaemic control compared with standard dietary advice. Educating women to consume a diet consistent with evidence-based recommendations for the dietary management of GDM [40] was chosen as the comparator group as it is safe for participants and this study aims to evaluate whether the interventions are more effective than usual care.

**Fig. 1:**
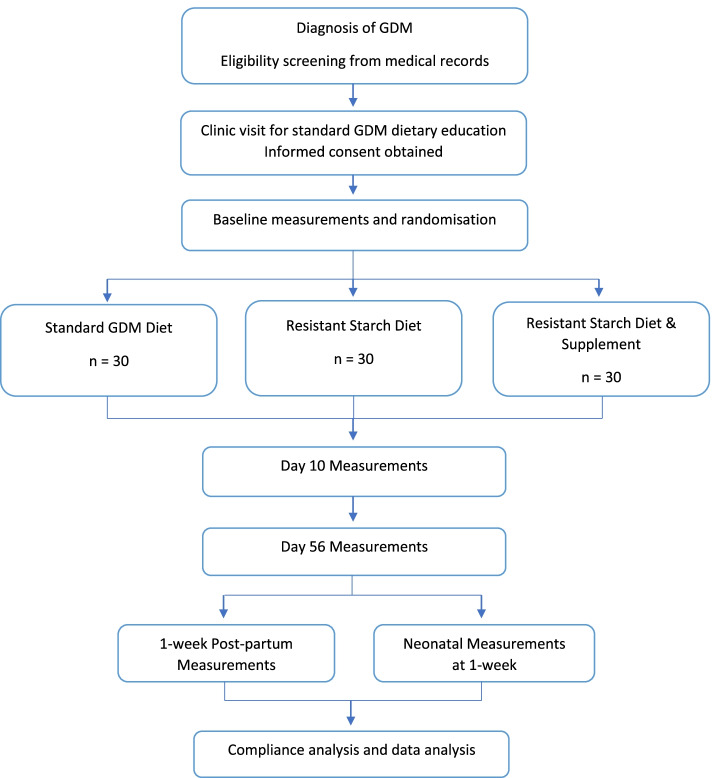
Flow diagram of study design, participant recruitment and journey. *Note:* GDM = Gestational Diabetes Mellitus

Participants will enter the trial at diagnosis with GDM between 24 and 30 weeks of gestation. Informed written consent will be obtained by the Principal Investigator (PI) prior to randomisation into one of three dietary treatment groups – standard dietary treatment for GDM (Control), a high RS diet (RS Diet) or a high RS diet plus an RS supplement (RS Supp). The dietary intervention will continue until delivery. Measurements will be taken at Baseline over Days 1–3 (where they will be between 24 to 36-weeks’ gestation), Day 10, Day 56 (approximately 36-weeks’ gestation) and 1-week after delivery (Fig. 1).

### Study population

Participants will be women who are newly diagnosed with GDM and plan to deliver their baby at a tertiary hospital in Western Australia, where all study visits and routine antenatal care will be undertaken. Inclusion criteria include women diagnosed with GDM through a routine 75 g Oral Glucose Tolerance Test (OGTT) between 24 and 30 weeks of pregnancy and ≥ 18 years of age. A diagnosis of GDM is made using the International Association of Diabetes in Pregnancy Study Groups (IADPSG) diagnostic criteria of one or more values reaching the following levels – Fasting glucose ≥5.1 mmol/L, 1-h ≥ 10.0 mmol/L, 2-h ≥ 8.5 mmol/L [41].

Participants will be excluded if they have an early diagnosis of GDM before 24 weeks; Overt Diabetes in Pregnancy; Type 1 Diabetes; T2DM; poorly controlled hypothyroidism; Graves’ Disease; twin pregnancy; breastfeeding; vegetarian; vegan; irritable bowel syndrome; inflammatory bowel disease; previous bariatric surgery; history of an eating disorder; allergy to adhesives; antibiotic use in the past 3 months; use of steroids, antipsychotics, metformin, laxatives, fibre supplements or probiotic supplement; any major medical disorder; any psychosocial issues likely to impact on ability to adhere to study protocol.

Demographic information will be collected from the digital medical record (DMR) and participants will complete a demographic questionnaire at Baseline to obtain information on ethnicity, medical and obstetric history, medication and dietary supplement usage.

### Sample size

In a study by Asemi et al. [42], the average woman with GDM has a mean FBG of 5.175 ± 0.86 mmol/L. In RS supplementation trials, a difference in FBG as large as 0.4 mmol/L has been observed between control and RS supplemented groups [38]. This study aims to demonstrate a reduction in FBG of 0.3 mmol/L, which corresponds to a small to medium Cohen’s effect size (*d* = 0.35). Based on a repeated-measures design with three groups (Control, RS Diet, RS Supp) and three time points (Baseline, Day 10 and approximately Day 56), a minimum sample size of 69 (i.e., 23 per group) is required to detect a small to medium within-between interaction effect (Cohen’s *f* = 0.175) at 80% power and 5% level of significance. Allowing for an attrition rate of around 30%, the final total sample size required is 90 (i.e., 30 per group). Women who commence insulin therapy will remain in the study.

### Recruitment

Women with newly diagnosed GDM who are potentially eligible to participate in the study will be given a study flyer. When attending clinic for standard GDM education, the Principal Investigator (PI) will explain the study and screen for eligibility, then invite eligible women to participate and provide written consent. The Participant Information Letter is provided in Supplement 1 and Participant Consent form in Supplement 2. Consent for data to be collected on the neonate will also be obtained.

Participant retention will be supported through undertaking study requirements at routine antenatal visits, complimentary parking, text reminders, supportive telephone contact from the PI between widely spaced appointments, and regular contact with the obstetric and midwifery team. Participants who withdraw consent to provide stool and urine samples will be given the option of providing fasting glucose samples.

### Randomisation

Stratified randomisation of participants based on pre-pregnancy Body Mass Index (BMI) category will be utilised. BMI categories are Healthy (BMI 18.5–24.9 kg/m^2^), Overweight (BMI 25–29.9 kg/m^2^) and Obese (BMI ≥ 30 kg/m^2^). A computer-generated random sequence was created by a statistician and repeating blocks of three groups per three BMI categories were used to generate group allocation order. Eligible participants will be randomised by the PI to the next predetermined group, sequentially as consented. Neither the participants nor the investigators will or can be blinded to the treatment allocated.

### Intervention

#### Dietary intervention

All women will receive standard GDM dietary advice in line with evidence-based guidelines [2, 40, 43, 44]. Women will be encouraged to consume a minimum carbohydrate intake of 175 g per day, from mostly low glycaemic sources, distributed across the day over three meals and three snacks. The dietary advice will promote a low saturated fat intake and higher consumption of vegetables, fruit, dairy and whole grains.

After Baseline data are collected, women who are randomised to either of the two dietary intervention groups (RS Diet & RS Supp) will receive additional dietary education on consuming a high RS diet commencing on Day 3. All RS dietary education will be conducted by the PI who is an experienced Accredited Practising Dietitian (APD). A standardised teaching plan (Supplement 3) and education materials will be utilised (Supplements 4 & 5). Participants will be provided with written material including a Gut Feeling cookbook [45] and sample menus, as well as some non-perishable samples of high RS foods to allow for immediate adoption of the diet. Dietary RS will be measured at Baseline, Day 10 and Day 56 using 3-day weighed food records (Supplement 6). Evidence of compliance with the high RS diet will also be monitored via urine samples for metabolomic analysis and change in stool microbiota at the same timepoints. Participants will be asked to continue the high RS diet until the delivery of their child.

The RS education tools and strategies have been piloted in a non-pregnant population and achieved a median increase in dietary RS intake of ≥6.6 g RS per day (unpublished data). The typical intake of RS in Australian women aged 19–44 years has been estimated to range from 2.9–8.3 g per day [46]. Similarly, recent data from the United States estimated the mean daily intake of RS to be 1.9 g per 1000 kcal for women of this age [47], which would equate to 3.8–5.7 g per day.

#### RS supplement

The RS Supp group will consume an RS supplement of high-amylose maize (HAMS) type 2 resistant starch. Participants will be given a 600 g tub of HAMS each fortnight, along with a 40 ml scoop. They will be instructed and provided with written material (Supplement 7) on how to prepare and incorporate the RS supplement into cool fluids or foods in their diet. As with all low-digestible carbohydrates, gastrointestinal (GI) discomfort is a known side effect of rapid introduction of RS, therefore, participants will introduce HAMS over a two-day adjustment period. They will be instructed to consume 1 scoop (20 g) per day for 2 days, taking half in the morning and the rest at night. Then, increasing to a dose of 1 scoop (20 g) morning and night for the remainder of their pregnancy. The RS supplement contains 40% RS type 2. The final amount of RS in the 40 g of HAMS per day will be 16 g, which is below the level of 45 g per day of RS supplementation that is known to be tolerated with minimal side effects, most of which are flatulence [48]. Intakes of up to 80 g RS per day have been tolerated without diarrhoea [48]. Those in the high RS diet plus RS supplement are unlikely to achieve this amount. Consumption and compliance of the RS supplement will be monitored by weight of unconsumed HAMS returned fortnightly. Participants will use a daily RS supplement diary to record intake as an additional measure of compliance. We have shown in a recent 2-week feasibility study, 100% compliance with consumption of the RS supplement and minimal GI side effects in 10 female participants with prediabetes or T2DM (unpublished). GI tolerance of the supplement will be measured via a daily bowel symptom diary for the first 10 days and Days 47 to 56.

Participants can withdraw from the study at any time for any reason. Participants will be withdrawn by the investigator if they commence antibiotics, steroids, metformin or if they can no longer comply with the study’s schedule of assessments (Table 1).

**Table 1:** Participant measurements and collection summary

	Day 1	Day 3	Day 10	Day 28	Day 42	Day 56	Day 7 post-partum
Recruitment & consent	√						
Randomisation of subjects	√						
Attend clinic	√	√	√		√	√	
Self-monitoring blood glucose data collection		√	√			√	
FreeStyle Libre Pro application	√				√		
Questionnaires:							
medical Hx	√						
medication	√						
GI symptoms	√		√			√	
Quality of Life SF36	√		√			√	
Weight measured	√		√			√	
3-day weighed food records collected		√	√			√	
Bowel symptoms, RS supplement, medication & exercise diary collected		√	√			√	
H_2_ breath test		√	√			√	
Fasting Bloods			√			√	
Maternal stool samples		√	√			√	√
Neonatal stool sample							√
Maternal urine sample		√	√			√	
Neonatal urine sample							√
Breastmilk sample							√
Study instructions and materials							
RS diet education		√					
supplement regime if applicable		√					
Reminder phone call or text				√			
Assessment of Medical Records for							√
adverse outcomes							
delivery method							
birth weight, length							
feeding method							
NICU admission							
length of stay							

*Note: RS* Resistant starch, *NICU* Neonatal Intensive Care Unit

### Primary outcome

#### Fasting blood glucose

The primary outcome of this study will be a reduction in median FBG from Baseline, within and between groups. Baseline FBG will be recorded from the diagnostic OGTT results [41]. Subsequent venous blood samples will be collected after an overnight fast at Day 10 and Day 56 and analysed for FBG following protocols from the National Association of Testing Laboratories. FBG within treatment targets (< 5.1 mmol/L) [49] is the measure of glycaemic control that has been most difficult to achieve with standard GDM diet and lifestyle interventions and therefore the variable most likely to indicate the need for medical therapy, such as insulin [50]. If FBGL below 5.1 mmol/L is not achieved by Day 10, insulin therapy will commence. Participants treated with insulin will remain in the study.

Routinely women will receive self-monitoring of blood glucose (SMBG) education. Participants will be provided with an SMBG meter and instructed on measuring blood glucose levels four times daily (FBG and 2-h post-prandially). The SMBG meters used will be either Contour Next (Ascensia Diabetes Care, Switzerland), Accu-Chek Guide (Roche Diabetes Care, Switzerland) or One Touch Verio (LifeScan, USA) and meet International Organization for Standardization (ISO) standards for accuracy. Data from these models will be uploaded via the Diasend Uploader (Glooko, USA). FBG will be monitored on the first 2 days and averaged to provide a Baseline measure. This will be compared to the moving averages over the subsequent 8 days of testing, and then again between Days 48 and 56, across the three dietary groups (Control, RS Diet and RS Supp) using mixed model ANOVA with group-by-time interaction.

### Secondary outcomes

#### Post-prandial glucose

Post-prandial blood glucose (PPBG) will be measured by participants using SMBG three times per day for three consecutive days. The change in mean 2-h PPBG and frequency of PPBG levels above the target for each mealtime will be calculated and compared within and between groups at Baseline (Day 1–3), Days 8–10 and Days 54–56. Two-hour PPBG excursions of 6.7 mmol/L or more are considered above the target for optimal pregnancy outcomes [49]. Elevated PPBG are associated with preeclampsia, caesarean section delivery, large for gestational age (LGA), neonatal hypoglycaemia [6] and childhood glucose and IR [10].

#### Time in range for glucose

Participants will be provided with and trained in the use of, a FreeStyle Libre Pro glucose sensor. On Day 1 and Day 42, using aseptic technique and following the manufacturer’s procedure, one FreeStyle Libre Pro glucose sensor (Abbott Diabetes Care, California, USA) will be applied to the mid-triceps area of each participant using a spring-loaded application device supplied with the sensor. A fine, flexible, 5 mm cannula extends from the sensor into the interstitial fluid and the sensor is secured on the skin via an attached adhesive pad. The sensors can be worn in the shower, whilst swimming or exercising. The sensors will be electronically paired with a FreeStyle Libre Pro reader through which the glucose data can be downloaded. The sensors continuously provide interstitial fluid glucose data every 15 min for 14 days and will allow for more accurate assessment of FBG, 2-h PPBG, frequency of 2-h PPBG levels elevated to ≥6.7 mmol/L and time in range (TIR) of optimal blood glucose levels of 3.5–7.8 mmol/L [51]. FreeStyle Libre glucose sensors have been validated in pregnancy against SMBG and found to be safe, accurate and acceptable to users [52]. Ethics approval has been obtained for use of FreeStyle Libre sensors in this study, however, funding for this is to be secured.

#### Requirement for insulin treatment

Data from the participants’ DMR will determine the percentage of women requiring insulin to control blood glucose levels at Day 10 and Day 56 compared to the control group. Participants who require insulin will remain in the study and all samples will be collected. The commencement of insulin to manage glycaemia requires more health care monitoring and intervention, adding burden to the woman and the health care system [18].

#### Microbiota

##### Maternal gut microbiota

All participants will receive written information (Supplement 8) and be instructed on the procedure for collection and storage of the first stool passed in the day at Baseline, Day 10, Day 56- and one-week post-partum. Each participant will be provided with a cooler and ice bricks for storage and transportation of stool samples. Upon receipt, stools samples will be weighed to the nearest 0.1 g on an electric balance (A&D, Japan) then immediately stored in a − 80 °C freezer. Stools will be thawed at 4 °C then homogenized and aliquoted, then restored at − 80 °C until analysed for microbial composition and their SCFA metabolites. This procedure will be repeated for each time point.

Microbial analyses will be performed at the Western Australian Human Microbiome Collaboration Centre at Curtin University. DNA will be extracted using the QIAamp PowerFecal Pro DNA kit (Qiagen). Microbiome signatures will be generated using the Illumina MiSeq platform barcoded V4 primer (515–806) targeting a hypervariable region of the 16S rRNA gene. Polymerase chain reaction free (PCR-free) ligation protocol will be deployed for library building. Samples will be sequenced to a depth of a minimum 20,000 reads, which is sufficient to identify microbes to a genus/species level. Quality control samples and mock communities will be included in the analysis from sample collection to sequence analysis. Sequence read quality will initially be assessed with FastQC before demultiplexing and pre-processing by GHAPv2, an in-house tool. Cutadapt [53] will be used for the removal of all non-biological sequences. DADA2 [54] will then be used for quality filtering, error correction, amplicon sequence variants (ASVs) picking. A trained Naïve Bayes classifier will then assign ASVs to genus/species against a curated database of microbial reference sequences such as the Ribosomal Database Project (RDP) [55] or Genome Taxonomy Database [56].

An increase in RS consumption is known to alter the gut microbiota in non-GDM populations [37]. An alteration from Baseline in stool microbial composition in RS groups towards a symbiotic composition compared with the control group will indicate that the increased RS consumption affects the microbiota of a woman with GDM and would indicate compliance with the RS dietary intervention.

##### Neonatal gut microbiota

A neonatal stool sample will be collected one-week post-partum for microbial analysis as above. These will be used to evaluate the effect of RS supplementation on microbiome seeding of the infants in relation to the maternal microbial composition. The neonatal sample will be collected and stored by the mother at home as per a standard procedure that will be provided with the stool sample kits before discharge (Supplement 9). In brief, the lid of the stool sample pot will contain an integrated scoop that is used to collect the majority of the neonate’s stool sample from the nappy. The sample pot will be capped with the filled scoop, placed immediately into a cooler lined with ice bricks and delivered to the hospital within 24-h. It will be weighed and stored at − 80 °C immediately.

##### Breast milk microbiota

If the mother has chosen to breastfeed, a 10 ml breast milk sample will be collected at one-week post-partum for microbial analysis. A standard procedure and collection tubes will be provided before discharge (Supplement 10). Briefly, after breastfeeding her infant, washing hands with soapy water and donning gloves, the mother will express breastmilk into two 5 ml sterile tubes from the same breast by hand or using a sterilised breast pump. Approximately 3–4 ml will be collected in each tube. Breastmilk samples will be frozen at home then delivered to the hospital in a cooler with ice bricks and immediately stored at − 80 °C until analysis for microbial composition at the Western Australian Human Microbiome Collaboration Centre at Curtin University. The microbiota of breast milk is postulated to be a determinant of the neonatal microbiota [57].

#### Faecal SCFA

The maternal stool samples collected, processed and stored as outlined above at Baseline, Day 10, Day 56 and one-week post-partum will be analysed for concentrations of SCFA (acetate, butyrate and propionate). The method of analysis will use gas chromatography and mass spectrometry as detailed by Stinson, Boyce [58]. An increase from Baseline in mean SCFA is a marker of increased gut microbiota fermentation. SCFA are thought to be the primary metabolites by which the microbiota affects glycaemic control [19–21, 59–62]. Neonatal stool samples collected at one-week post-partum will also be analysed for SCFA content using the above method.

#### Anthropometric measurements

Maternal height will be measured (to the nearest 0.1 cm) by a trained nurse at Baseline using a stadiometer (Seca, Germany). Weight will be measured (to the nearest 0.1 kg) at Baseline, Day 10 and Day 56 using scales (A & D Medical, Japan). Excessive gestational weight gain compromises maternal glycaemic control and therefore it is important to ensure this does not differ between groups.

The birth weight of the neonates’ will be measured to the nearest 5 g by a trained midwife using the scales of a Panda Warmer (General Electric Healthcare, USA). Length at birth will be measured to the nearest centimetre using a metric tape measure. Anthropometric data will be collected from the DMR of the neonates to determine the mean birth weight, Ponderal Index [weight (g) ÷ length (cm)^3^] and percentage of LGA or macrosomic neonates of the intervention groups (RS Diet & RS Supp) compared to the control group. High birth weights are associated with shoulder dystocia, caesarean section delivery, post-partum haemorrhage, childhood obesity, and insulin resistance [63] and therefore one of the main complications of GDM that all treatments aim to improve.

#### Gastrointestinal tolerance

##### Bowel symptoms questionnaire

Mild gastrointestinal side effects are expected with a high consumption of RS. A GI symptoms questionnaire adapted from Francis [64] will be administered at Baseline, Day 10 and Day 56 (Supplement 11). The questionnaire contains a visual analogue scale between 0 and 100 for each of four symptoms, generating a total score of up to 400. Participant responses will be measured manually from the zero mark by the PI and converted to a score out of 100. Scores will be used to evaluate the effect of the interventions on GI comfort and function.

##### Bowel symptoms record

Participants will be asked to keep a bowel symptoms diary at Baseline (Day 1 to 3), Days 8 to 10 and Days 54 to 56 to assess tolerance of the diet and supplement (Supplement 12). This will be returned at the Day 10 and Day 56 study visits. They will record the frequency of bowel movements, rate each movement on a scale of 1 to 7 for consistency using the Bristol Stool Chart [65] and ease of stool passage on a scale of 1–5 where 1 = very easy and 5 = very difficult [66]. The diary includes a subjective scoring of symptoms of flatulence, borborygmus, abdominal cramping and distention, nausea, diarrhoea, constipation. Scores will be chosen from a scale of 0 to 3, where 0 is for no symptoms beyond their normal and 3 is a rating of severe symptoms, an evaluation method used previously by other researchers [67]. The average composite score for individuals’ first 2 days prior to the intervention will be compared to the average score for the second week of the study to establish whether there is a statistically significant difference within individuals and between groups. Scores for individual symptoms will be similarly complied to compare differences within individuals and between groups. This scoring system will also be used as one mechanism for identifying and rating adverse events (AE), along with participants self-reporting of AE. A score of 3 (severe) will be considered an AE and reported in the publications resulting from this trial. A small feasibility study in women with prediabetes or diet controlled T2DM (*n* = 10) trialled the high RS diet and the RS supplement and they were well tolerated (unpublished data). Participants will be encouraged to report any AE to the PI who will escalate to the appropriate health care professional and human ethics committee immediately.

#### Resistant Starch intake

##### Food records

All participants will be instructed by the APD to keep a 3-day weighed food record at Baseline (Days 1–3), Days 8–10 and Days 54–56 for assessment of mean RS intake (Supplement 6). Food records will be collected at the next study visit. Alternatively, participants can choose to log their food intake using the Research Food Diary app (Xyris Software, Queensland, Australia). To aid in the accurate measurement of food consumed, kitchen scales (Propert, China), metric measuring cups and measuring spoons will be lent to participants. Nutrient analysis of food records will be completed using FoodWorks 10 (Xyris Software, Queensland, Australia). Databases on RS content of foods are limited and RS content varies widely depending on cultivars, growing conditions, country of origin, food processing, preparation and storage methods, and methods of analysis [61]. Published RS values for individual foods vary widely. Therefore, a database created from the minimum and maximum RS values published by various authors [46, 68–70] has been created by Edith Cowan University researchers and will be utilised in this study to calculate RS intake. This database has previously been used for nutrition research [71].

##### RS supplement consumption diary

Participants will be provided with a supplement diary as one measure of compliance with the RS regime (Supplement 13). Compliance with the study protocol will be achieved if at least 80% of the RS supplement doses have been recorded as consumed over the study period.

##### RS supplement returned

Weighed portions of the RS supplement will be provided to participants fortnightly. Unconsumed RS supplement will be returned by participants and weighed on kitchen scales (Propert, China), to assess the percentage consumed and subsequent compliance with the RS consumption target.

##### Breath H_2_

A handheld hydrogen breath analyser (H_2_ Check, MD Diagnostics Ltd., UK) will be used to assess change in H_2_ production with the RS interventions. This will also be used as a measure of compliance with the RS diet and RS supplement. Breath H_2_ measurements will be taken Day 3, Day 10 and Day 56. Ethics approval has been obtained to collect Breath H_2_ data, however, funding has to be secured.

#### Health-related quality of life

Participants will complete the RAND 36-Item Health Survey 1.0 (*SF-36*) [72] at Baseline and Day 10 and Day 56. Scoring will be completed and standardised with Australian reference ranges using the methods and data from the Australian Longitudinal Study of Women’s Health [73]. Scores will determine if the health-related quality of life is maintained across nine health domains during the intervention.

#### Metabolomics

##### Blood (maternal)

Fasting maternal blood samples will be collected on Day 10 and Day 56. Phlebotomy and processing will be performed by a registered pathology service following their standard operating procedures for handling blood. In addition to a fasting glucose test, 8.5 ml of blood will be drawn into a serum tube, centrifuged, 1 ml aliquots transferred into 4 tubes and stored in a − 80 °C freezer for analysis of SCFA content, lipids and metabolomics.

##### Urine (maternal and neonatal)

First void maternal urine samples will be collected as per a standard procedure (Supplement 14) at Baseline, Day 10 and Day 56 and stored at − 80 °C until metabolomic analysis can be performed at the Australian National Phenome Centre, Western Australia. Urinary metabolomic markers of foods consumed will be used to assess compliance with the consumption of high RS foods by the RS intervention groups. Additionally, metabolomic analysis will seek to identify additional biochemical markers that enable a better understanding of the systems biology effects of the intervention.

A neonatal urine sample for metabolomic analysis will be collected by the mother 1-week post-partum as per a standard procedure that will be provided with the stool sample kits before discharge (Supplement 15).

#### Maternal and neonatal outcomes and cost of antenatal care

Maternal and neonatal outcomes data will be collected from the DMR after discharge. Diagnosis Related Group (DRG) [74] data will be collected for calculation of hospital costs. Maternal outcome data collection will include the number of ANC clinic visits, Maternal Foetal Assessment Unit visits, antenatal admissions, delivery method, post-partum maternal length of stay, and feeding method on discharge.

After discharge, data on admission to the Neonatal Intensive Care Unit (NICU), reason for admission and length of stay will be collected from the DMR of the infant to determine the frequency of admissions to NICU in the intervention groups compared to the control group. NICU admission for neonatal hypoglycaemia or respiratory support is a known complication in babies of GDM mothers and is one of our secondary outcomes for the study. There is a linear relationship between glycaemia and admissions to NICU in offspring of women with GDM [6]. We will record this outcome and collect neonatal samples as and when the baby’s clinical condition allows. We accept that research on pregnant women raises particular safety concerns. It is noteworthy that our intervention is safe and we do not expect serious adverse events as a direct result of the intervention. We expect neonatal deaths to be not different from the general population.

### Data management

Individually identifiable data will be coded as soon as possible. Data and codes will be kept in separate lockable filing cabinets within a swipe card accessible office and access to the data will be restricted to the research team. Electronic data will be kept in a de-identified format and stored on a password-protected computer or secure server and for a minimum of 25 years. At the end of the retention period, data files and any hard copy source data will be deleted/shredded as per the South Metropolitan Health Service and Edith Cowan University data management requirements. Data collected on participants who later withdraw will be used in analysis if required unless consent to use it has been withdrawn.

The trial investigators/institutions will permit trial-related monitoring, audits, and regulatory inspections, providing direct access to source data/documents. This may include, but not limited to, review by Human Research Ethics Committees and institutional governance review bodies.

### Statistical analyses

Baseline demographic and outcome variables will be described and compared for differences between groups. Continuous variables will be described as mean ± standard deviation (SD) and nominal and ordinal variables as frequencies and proportions. All continuous variables will be examined for normality using the Shapiro-Wilk test. Descriptive statistics for non-normal continuous variables will be presented as median ± interquartile range (IQR).

FBG and PPBG will be monitored on the first 2 days and averaged to provide a Baseline measure of each outcome. These will be compared to those measured over the subsequent 8 days, and then again between Days 48 and 56, across the three dietary groups (Control, RS Diet and RS Supp) using linear mixed modelling with group-by-time interaction. Analysis will assess if a 0.3 mmol/L reduction is achieved. Demographic variables such as age, pre-pregnancy BMI, parity and ethnicity will be adjusted in the model. Statistical analyses will be performed using SPSS® (Statistical Package for Social Sciences, version 27 for Windows).

The number of PPBG excursions experienced by a participant per day between Days 4 to 10 will be examined and compared relative to the number determined at their Baseline over the first 2 days of the usual care diet. Generalised Mixed Modelling will be utilised to assess whether a dietary change reduces the frequency of PPBG excursions as compared with norm with adjustments for relevant demographic variables.

Different R packages and PRIMER 7 (Quest Research, NZ), a non-parametric statistical software package, will be used to assess change within and between groups for microbial composition. Distance Based Linear Modelling (DistLM) will be used to look for a correlation between change in stool microbiota or SCFA & measures of glycaemia.

Direct and indirect costs savings related to any reduction in insulin treatment or improvements in maternal and neonatal outcomes will be calculated from DRG codes, Weighted Activity Units (WAU) and the National Efficient Price (NEP) and reported [74].

Missing data will be treated in statistical analysis as missing and coded as 999 or left blank, so as not to affect the accuracy of the analysis. Prior to any statistical analysis, all data will be explored for outliers. Any outliers found will be cross-checked with the source file. Any true outliers will be checked with the clinician before being removed/left in the dataset.

## Discussion

GDM is prevalent and optimal glycaemic control offers health benefits to the mother and child [2]. Current dietary strategies have proven positive outcomes [5, 40] but are not effective enough for more than half of women to avoid insulin therapy [14]. Additionally, women post-GDM and their offspring remain at greater risk of metabolic health problems over their lifetimes [2]. A cost-effective lifestyle solution to further improve glycaemic control and minimise the requirement for medication is necessary to both achieve better maternal and neonatal outcomes and to reduce the burden on health care systems [18]. Therefore, novel dietary strategies are required.

Gut dysbiosis has been reported as a feature of GDM pregnancies [21–23] and it is established that dietary intake of fermentable fibres, such as RS, changes the gut microbiota and metabolome [39, 75]. RS supplementation has also been shown to improve glycaemic control in prediabetes and type 2 diabetes [28], with the likely mechanism via SCFA produced during the fermentation of RS by the gut microbiota [20]. The three main SCFA’s acetate, propionate and butyrate have all been found to play an essential role in maintaining a healthy gut, insulin resistance and insulin sensitivity. Butyrate enhances gut wall integrity, reducing gut permeability to endotoxins thereby lessening adipose tissue inflammation and IR [19–21, 59, 60]. Butyrate is also thought to stimulate colonic L-cells to release glucagon-like peptide-1 (GLP-1), and other gut hormones, which improve glucose homeostasis through increasing glucose-dependent insulin secretion, suppressing glucagon secretion, slowing gastric emptying and reducing appetite [20, 76, 77]. Acetate and propionate are directly associated with improved insulin sensitivity of adipose and skeletal muscle cells [61, 62].

Studies have characterised gut microbiome changes in the GDM population [21, 22, 35, 78, 79], assessed the effect of dietary manipulations on the maternal gut microbiome [35, 80, 81] or the relationship between the microbiome and glycaemic control in GDM [22, 23, 78, 79, 82]. However, very few randomised control trials have examined all these parameters in the same study, using a diet intervention to manipulate the gut microbiome to improve glycaemia and achieve better outcomes in a GDM population. For instance, Mokkala et al. [81] showed that fish oil and probiotic supplements were not effective in altering the microbiome or serum glucose in overweight and obese GDM women. To our knowledge, there are no studies that have investigated the relationship between dietary RS intake, the gut microbiome, glycaemic control and maternal and foetal outcomes in pregnancies affected by GDM, therefore, this needs to be explored as a lifestyle and economical approach to management.

This open-label, parallel-group design study will build on the emerging relationship between dietary RS, the gut microbiome and improved blood glucose levels [24–28]. It will not only characterise the changes to the maternal gut microbiota in response to a higher dietary intake of RS but proceed to identify any correlation between these microbial changes and improvements in glycaemic control. Again, only a few studies have done this in non-pregnant populations. Additionally, it will investigate whether maternal microbial changes in response to RS can alter the microbiota of the neonate, possibly inoculating the next generation with a more favourable microbiota. Also unique to this study is the assessment of whether a high dietary intake of RS from whole foods alone can improve glycaemic control in GDM, or if an RS supplement is required to achieve a positive result.

A limitation of this study is that food will not be provided to the participants which may limit compliance with a high RS diet. However, a positive result would indicate that this dietary intervention is a practical and achievable intervention to incorporate into GDM management strategies. Periodic assessment of glucose is also a limitation of the study as it only estimates overall glycaemic control but is the most frequently used method in the management of GDM, whereas continuous blood glucose monitoring would assess the overall exposure of the foetus to glucose. Funding for continuous blood glucose sensors is not yet available, therefore, an opportunity to identify any overall glycaemic benefit through time-in-range values may be missed. The study is adequately powered to detect improvements in FBG and microbial changes (based on changes in faecal SCFA) but not for other outcomes such as rates of LGA, macrosomia, neonatal hypoglycaemia and NICU admissions.

This will be the first study to evaluate RS intervention in GDM management. If a high dietary intake of RS is shown to favourably alter the GDM microbiome and results in improved glycaemic control, this will expand the dietary interventions available to manage GDM without pharmacotherapy, reducing the burden on the mother and the healthcare system. If a more favourable maternal gut microbiota is found to persist post-partum and is transferred to the neonate, longer-term health outcomes for mother and child may be enhanced.

## Supplementary Information

**Additional file 1.**

**Additional file 2.**

**Additional file 3.**

**Additional file 4.**

**Additional file 5.**

**Additional file 6.**

**Additional file 7.**

**Additional file 8.**

**Additional file 9.**

**Additional file 10.**

**Additional file 11.**

**Additional file 12.**

**Additional file 13.**

**Additional file 14.**

**Additional file 15.**

**Additional file 16.**

## Data Availability

The datasets generated in this study will be made available in the Edith Cowan University repository and will be available from the corresponding author on reasonable request.

## Abbreviations

AE: Adverse events
APD: Accredited Practising Dietitian
BMI: Body Mass Index
DMR: Digital medical record
DRG: Diagnostic Related Group
FBG: Fasting blood glucose
GDM: Gestational diabetes mellitus
H_2_: Hydrogen
HAMS: High-amylose maize starch
IR: Insulin resistance
LGA: Large for gestational age
NICU: Neonatal Intensive Care Unit
OGTT: Oral glucose tolerance test
PI: Principal investigator
PPBG: Post-prandial blood glucose
RS: Resistant starch
SCFA: Short-chain fatty acids
SF-36: RAND 36-Item Health Survey 1.0
SMBG: Self-monitoring of blood glucose
T2DM: Type 2 Diabetes Mellitus
