# Supplementary material for: Does a high dietary intake of resistant starch affect glycaemic control and alter the gut microbiome in women with gestational diabetes? A randomised control trial protocol
Source: BMC Pregnancy Childbirth. 2022 Jan 18;22:46. doi: 10.1186/s12884-021-04366-4 (PMC8764780; doi:10.1186/s12884-021-04366-4)
Supplement: Supplementary file 1 — Additional file 1. [file 12884_2021_4366_MOESM1_ESM.docx]

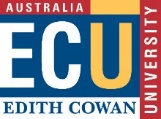
Supplement 1

**Participant Information Letter**

**Project Title:** *The Effect of Dietary Resistant Starch on Maternal Glycaemia and Gut Microbiome in Gestational Diabetes*

**Ethics Approval Number:**

**Principal Investigator:**

**An invitation to participate in research**

You are invited to participate in a research project titled *The Effect of Dietary Resistant Starch on Maternal Glycaemia and Gut Microbiome in Gestational Diabetes.* It seeks to establish if consuming a diet high in resistant starch can improve your blood glucose levels and result in better health outcomes for yourself and your baby.

You are being asked to take part in this project because you have recently been diagnosed with Gestational Diabetes.

Please read this information carefully. Ask questions about anything that you do not understand or want to know more about. Before deciding whether or not to take part, you might want to talk about it with a relative or friend.

If you decide you want to take part in the research project, you will be asked to sign the consent section. By signing it you are telling us that you:

- Understand what you have read;
- Consent to take part in the research project;
- Consent to be involved in the research described;
- Consent to the use of your personal information as described.

**What is this project about?**

This project aims to investigate whether a high dietary intake of resistant starch (RS) can improve blood glucose control when compared with the usual diet prescribed to manage gestational diabetes. Importantly, it will also look at whether this diet changes your gut microbiome and if this is the reason blood glucose levels are improved. The study will be conducted on women with newly diagnosed gestational diabetes who attend _______ Hospital for their antenatal care. It is hoped that a positive result from this study will add to the evidence required to enhance current dietary recommendations for the management of gestational diabetes.

A grant has been received from ______________.

**What does my participation involve?**

Your participation in this research project will involve the following:

If you qualify for the study, you will be asked to stay at the hospital for an extra 30-60 minutes after your gestational diabetes education session. You will be randomly allocated to one of three groups for the remainder of your pregnancy:

1. A group who will be asked to follow the standard dietary recommendations to manage gestational diabetes;
2. A group who will be instructed on how to consume a diet that is high in resistant starch, which also fits with the standard dietary recommendations to manage gestational diabetes;
3. A group who will be instructed on how to consume a diet high in resistant starch and asked to consume a powdered food supplement of resistant starch, which is stirred into cold food or drinks.

Note that none of the different treatments has been proven to be better than another and you will not have a choice of which group you are allocated to.

As well as doing the finger-prick blood glucose testing that you will be taught, your blood glucose levels will be measured continuously for two periods of two weeks by utilising ‘flash’ glucose monitoring technology. This is a small, white, plastic disc which will be attached to the back of your upper arm. The glucose levels will be measured by a very short, fine plastic filament which will extend from the disc into your skin. This sensor only takes a few seconds to apply using a spring-loaded device and is usually painless. The blood glucose results will be transferred to a reading device as it is waved across the sensor. The data will be encrypted and uploaded to a secure cloud-based software program which is accessible by the Principal Investigator.

When you participate in the study you will also be asked to complete a series of questionnaires on topics including your medical history, medications, your bowel habits and your quality of life. Your height and weight will be measured. You will be asked to complete a diary of all the food and drinks that you consume for three short periods during the study. You will be asked to record details of your bowel habits over those same periods. You will also be asked to collect the following samples:

- Four stool samples during the study and another from your baby one week after birth,
- Three urine samples during the study and one from you baby one week after birth,
- One breastmilk sample one week after birth, if you choose to breastfeed.

**Details of the assessments**

1. Demographic and medical history questionnaire: This questionnaire will record your general health and demographic information.
2. Bodyweight: Your height and weight will be measured at the commencement of the study and collected from your antenatal appointment visit records.
3. Bowel symptom questionnaire: This questionnaire will allow the researcher to evaluate any change in your bowel habits between the beginning and end of the study.
4. Bowel symptom log: For three short periods during the study, you will be required to record any bowel symptoms encountered during these times. These records will be collected at the next study visit. They will help to identify if the changes to your resistant starch intake affect your bowel habits.
5. Exercise and medication logs: These will be used to assess other factors that may affect the study results. They will be collected at the next study visit.
6. 3 day-weighed food record (and resistant starch supplement consumption if applicable): You will be required to weigh and record (on a form provided to you) all food and drink consumed over the three recording periods. They will be collected at the next study visit. Alternatively, you will be able to record the food you consume on an Australian smartphone app called Research Food Diary. All data is stored on secure servers and protected from unauthorized access. You will be asked to share the food intake data that you collect on the app with the Principal Investigator when you return for study visits. Once shared, that data will be stored on a password protected computer within a swipe card accessed office.
7. FreeStyle Libre Pro blood glucose sensor: A sensor will be applied to your upper arm to measure your blood glucose levels continuously over 2-weeks. This will occur twice during the study period.
8. Blood tests: You will be asked to provide two fasting blood samples. These will be used to test your blood glucose at that time and a sample retained for analysis of other substances produced by your body.
9. Stool Collection: You will be provided with instructions on how to collect and store a stool sample. You will be provided with a cooler and ice bricks to store and transport each sample. You will be asked to collect your stools three times during your pregnancy and to collect a sample from yourself and your baby after discharge from hospital. Please be assured that all samples will be de-identified and sent to an external lab for analysis. None of the researchers or other staff involved in your care will see the samples, so your anonymity is assured.
10. Urine samples: You will be provided with instructions on how to collect and store urine samples. You will be asked to collect your urine three times during your pregnancy (Day 3 & Day 10 of the study and at 36 weeks’ of pregnancy) and to collect a sample from your baby one week after discharge from hospital.
11. Breast milk sample: If you choose to breast feed, you will be asked to collect a 10 ml sample of breast milk one week after discharge from hospital.
12. Hydrogen breath test: You will be asked to breathe into a breath analyser on three occasions.
13. You will receive 2-4 texts or phone calls from the Chief Investigator during the study to check your progress, remind you to comply with the study requirements and to confirm the appointment times of your 34 and 36-week antenatal visits.

|  | **Day 1** | **Day 3** | **Day 10** | **32 weeks pregnant** | **34 weeks pregnant** | **36 weeks pregnant** | **1 week after delivery** |
| --- | --- | --- | --- | --- | --- | --- | --- |
| Attend clinic  (1 extra visit) | **√** | **√**  extra visit | **√** |  | **√** | **√** | **√** |
| Questionnaires | **√** |  | **√** |  |  | **√** |  |
| 3-day Food diary | **√** |  | **√** |  |  | **√** |  |
| Bowel Symptom, Medication and Exercise Record | **√** |  | **√** |  |  | **√** |  |
| Weight | **√** |  | **√** |  |  | **√** |  |
| Glucose sensor applied | **√** |  |  |  | **√** |  |  |
| Start test diet |  | **√** |  |  |  |  |  |
| Blood sample |  |  | **√** |  |  | **√** |  |
| Stool sample |  | **√** | **√** |  |  | **√** | **√** |
| Baby’s stool sample |  |  |  |  |  |  | **√** |
| Urine sample |  | **√** | **√** |  |  | **√** |  |
| Baby’s urine sample |  |  |  |  |  |  | **√** |
| Breastmilk sample |  |  |  |  |  |  | **√** |
| Hydrogen breath test |  | **√** | **√** |  |  | **√** |  |
| Text/phone |  |  |  | **√** |  |  | **√** |

**Do I have to take part in this research project?**

No. Your participation in this research project is voluntary. If you do not wish to take part, you do not have to. If you decide to take part and later change your mind, you are able to withdraw (quit) at any time.

If you do decide to take part, you will be given this Participant Information Letter and Consent form to sign and you will be given a copy of the information letter to keep. Your decision to take part, or to take part and later withdraw, will not affect your relationship with the researchers or the team.

**What if I withdraw from this research project?**

If you decide to withdraw from this research project, please notify a member of the research team before you withdraw. A member of the research team will inform you if there are any special requirements linked to withdrawing. If you do withdraw, then with your agreement, the research team would like to retain and use any data that has already been collected. You will also be asked to sign a Withdrawal of Participation Form.

**What are the alternatives to participation?**

If you choose not to participate in this research, you will receive the same dietary advice and standard care that is routinely provided to women who are diagnosed with GDM at _____. You will still be asked to follow a diet, test your blood glucose levels frequently and attend regular appointments at the hospital where your blood glucose levels will be assessed.

**Can I have other treatments during this research project?**

Yes. You can have any treatment that you or your health care team deem necessary.

**Your privacy**

By signing the consent form, you consent to the research team collecting and using personal information about you or information about your health for the research project. Any information obtained in connection with this research project that can identify you will remain confidential. Individually identifiable data will be coded as soon as possible. Data and codes will be kept in separate lockable filing cabinets. Access to the data will be restricted to the research team. Electronic data will be kept in a deidentified format and stored on a password-protected computer or secure server and for a minimum of 25 years. Data files and any hard copy source data will be deleted/shredded as per the health service and university data management requirements.

Blood,stool, urine and breastmilk samples collected during this study will be identified only by codes and used solely for this study. Blood samples will be collected and processed by ___________, then stored for analysis at _________. Urine samples will be stored for analysis at ______. Stool and breastmilk samples will be sent to _______ for analysis. Spare samples will be retained for 10 years at -80˚C and then destroyed. Only trained staff from the facilities above will have access to the samples.

It is anticipated that the results of this research project will be published and/or presented in a variety of forums. A final report will be submitted to examiners and funding bodies. In any report, publication and/or presentation, information will be provided in such a way that you cannot be identified, except where requested for specific reasons, and then you will be asked to provide written consent.

In accordance with relevant Australian and/or Western Australian privacy and other relevant laws, you have the right to request access to the information about you that is collected and stored by the research team. You also have the right to request that any information with which you disagree be corrected. Please inform the research team member named at the end of this letter if you would like to access your information.

**Possible Benefits**

We cannot guarantee or promise that you will receive any benefits from this research, however, possible benefits may include better blood glucose control which could result in better health outcomes for yourself and your baby. This research may not provide benefit to you personally but may provide benefits for people with gestational diabetes in the future.

**Possible Risks and Risk Management Plan**

You may feel that some of the questions we ask are stressful or upsetting. If you do not wish to answer a question, you may skip it and go to the next question, or you may stop immediately. If you become upset or distressed as a result of your participation in the research project, the research team will be able to arrange for counselling or other appropriate support.

Consumption of very high amounts of dietary fibres such as resistant starch can cause mild gastrointestinal upset, most often temporary flatulence. To minimise discomfort, the resistant starch supplement will be introduced slowly over a two-day adaptation period. There is no evidence of any risk of a high resistant starch diet or supplement to unborn babies.

The glucose sensor application is usually painless but may cause very mild discomfort initially. It is stuck to the arm with an adhesive. People with allergies to adhesives or sticking plasters should not participate in this study. Mild skin irritation could still occur in the absence of an allergic reaction.

**What if new information arises during this research project?**

If new information arises about the benefits of dietary resistant starch on GDM during this study, it is unlikely to be enough evidence to change the standard dietary guidelines at this stage. Therefore, this study will continue and hope to contribute to the body of evidence. If any evidence arises that questions the safety of resistant starch in pregnancy, the research team will assess the risk and stop the study if required.

**Could this research project be stopped unexpectedly?**

In the unlikely event that the Principal Investigator is unable to continue to collect data, or if an unexpected risk to participants is identified, the research may be stopped.

**What happens when this research study is complete?**

We will advise you of the study outcomes via a one-page summary at the end of the study. We also intend to publish our results in research journals and present them at research conferences locally, nationally and internationally. Your name or any other identifying information will not be included in any of the publications or presentations.

Sharing research data creates opportunities to increase the pace of knowledge discovery and scientific progress. The data in this study could be shared with other parties and will be made available through the __________. De-identified data from this study may be used in future studies by this team.

**Has this research been approved?**

This research project has received the approval of ______________________.

**Contacts**

If you would like to discuss any aspect of this project, please contact the following people.

| **Principal Investigator** |  |
| --- | --- |
|  |  |
|  |  |
|  |  |

**If you have any concerns or complaints about the research project**

You may wish to talk to an independent person. You can contact:

|  |  |
| --- | --- |

If you would like to participate in this study, then please sign the Consent Form. Thank you.

Sincerely,

Principal Investigator
