# Supplementary material for: Does a high dietary intake of resistant starch affect glycaemic control and alter the gut microbiome in women with gestational diabetes? A randomised control trial protocol
Source: BMC Pregnancy Childbirth. 2022 Jan 18;22:46. doi: 10.1186/s12884-021-04366-4 (PMC8764780; doi:10.1186/s12884-021-04366-4)
Supplement: Supplementary file 2 — Additional file 2. [file 12884_2021_4366_MOESM2_ESM.docx]

Supplement 2


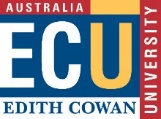
**Participant Consent Form**

**Student Project Title:** *The Effect of Dietary Resistant Starch on Maternal Glycaemia and Gut Microbiome in Gestational Diabetes*

**Approval Number:**

**Principal Investigator:**

I, __________________________________ have read the Participant Information Letter or someone has read it to me in a language that I understand. By signing this consent form, I acknowledge that:

- I understand what my involvement in this research will include
- data will be collected from the birth records of my baby
- my baby’s stool and urine samples will be analysed
- my blood, stools, urine and breastmilk will be analysed
- I have had any questions answered to my satisfaction
- I understand the risks of the research
- I freely agree to participate in this research project as described and understand that I am free to withdraw at any time during the study without prejudice
- I will have all identifiable information I provide treated as confidential
- the Principal Investigator will access my identifiable glucose sensor data via the Libre Link app. This encrypted data will only be visible to the Principal Investigator on a password protected computer; and
- de-identified data collected in this study may be used for future research purposes. You may withdraw consent for the data to be used at any time.

| Participant name: |  | | |
| --- | --- | --- | --- |
| Signature: |  | Date |  |

Approval to conduct this research has been provided by the ____________Human Research Ethics Committees, approval number ________, in accordance with its ethics review and approval procedures. If you have any concerns or complaints about the research project and wish to talk to an independent person, you may contact the Manager, _____________

**Form for Withdrawal of Participation**


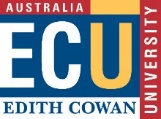


**Student Project Title:** *The Effect of Dietary Resistant Starch on Maternal Glycaemia and Gut Microbiome in Gestational Diabetes*

**Approval Number:**

**Principal Investigator:**

**Declaration by Participant**

I wish to withdraw from participation in the above research project and understand that such withdrawal will not affect my routine care, or my relationships with the researchers, Fiona Stanley Hospital or Edith Cowan University.

□ I give consent for any data or samples already collected to be used in this research

OR

□ I do not give consent for any data or sample already collected to be used in this research and request that all are destroyed.

|  | | | | | | | |
| --- | --- | --- | --- | --- | --- | --- | --- |
|  | Name of Participant (please print) | |  | |  |  |  |
|  | | | | | | | |
|  | Signature |  | | Date | |  |  |
|  | | | | | | | |

In the event that the participant’s decision to withdraw is communicated verbally, the Senior Researcher must provide a description of the circumstances below.

|  |
| --- |

**Declaration by Researcher^†^**

I have given a verbal explanation of the implications of withdrawal from the research project and I believe that the participant has understood that explanation.

|  | | | | | | |
| --- | --- | --- | --- | --- | --- | --- |
|  | Name of Researcher (please print) | |  | | |  |
|  | | | | | |  |
|  | Signature |  | | Date |  |  |
|  | | | | | | |

^†^ An appropriately qualified member of the research team must provide information concerning withdrawal from the research project.

Note: All parties signing the consent section must date their own signature.
