# Supplementary material for: Does a high dietary intake of resistant starch affect glycaemic control and alter the gut microbiome in women with gestational diabetes? A randomised control trial protocol
Source: BMC Pregnancy Childbirth. 2022 Jan 18;22:46. doi: 10.1186/s12884-021-04366-4 (PMC8764780; doi:10.1186/s12884-021-04366-4)
Supplement: Supplementary file 3 — Additional file 3. [file 12884_2021_4366_MOESM3_ESM.docx]

Supplement 3

| **Teaching Plan: High Resistant Starch (RS) Gestational Diabetes (GDM) Diet** | |
| --- | --- |
| **Learners Characteristics**  The following individual education session is targeted to the participants of the study entitled *The Effect of Dietary Resistant Starch on Maternal Glycaemia and Gut Microbiome in Gestational Diabetes.* They will be between 18 and 45 years of age, newly diagnosed with GDM and booked to deliver their baby at ______ Hospital. This is a culturally and socioeconomically diverse group, many of whom are very capable and motivated to learn to self-manage their GDM. It will be important to respect their prior knowledge and experience and modify the content of the teaching plan to suit the individual. They may have already researched GDM on the internet and will have just attended a GDM diet group education session but are unlikely to have much prior knowledge of high RS food sources. The education session needs to be succinct, logically organised, related back to what they have learned of the dietary management of GDM and broken down into steps that correspond to the study protocol. | |
| **Session Aim(s)**   - The participant will understand how they can consume a diet high in RS within the guidelines of a diet suitable for Gestational Diabetes | **Learning Objectives**  **At the completion of the session the client will be able to**   - Identify foods with a high RS content - Understand how much of the high RS foods they need to consume daily - Be able to articulate an example of a high RS breakfast, lunch, dinner and snacks that also fit with carbohydrate recommendations for GDM - Have a plan for what high RS foods they need to buy today to start implementing the diet immediately - If applicable, understand how much RS supplement they need to consume at each study time point and understand what foods to mix it into. |
| **Resources**   - Diagram/infographic - *High RS Shopping List* - *High RS Menus* - *Gut Feeling* Cookbook - *Tips for Using RS* | |

| **TIME** | **CONTENTS** | **FACILITATOR ACTIVITY** | **LEARNER ACTIVITY** | **RESOURCES** | **EVALUATION** |
| --- | --- | --- | --- | --- | --- |
| 1 min | Introduction | - Thank participant - Reassess commitment to participate in the study - Reflect on what a GDM diet tries to achieve and its limitations | - Active participation in discussion with educator |  | - Participant affirms that intend to follow the study protocol |
| 2 min | What do we think a high RS intake will achieve? | - Explain how RS is not digested, goes to colon, fermented by microbes, produce SCFA. Benefit of SFCA on gut integrity and insulin resistance. - Hope to see a reduction in Fasting BGLs, post-prandial BGLs and therefore reduced need for insulin - Hope to see better outcomes for mother and baby (PE, LGA, macrosomia, shoulder dystocia and NICU admission) - Expect to see a change to microbiota of mother and baby | - Discussion with participant about their understanding of concept | - Infographic of mechanism | - Participant can articulate that the high RS foods will change microbiota and that the study aims to see if that results in better GDM outcomes |
| 3 min | Which foods are high RS? |  | - Talk through shopping list of which foods she likes and might incorporate more of into her diet | - *High RS Shopping List* | - Agreement to purchase and consume more high RS foods |
| 10 mins | What kind of meals could they make? | - Discuss example meals from High RS Menus - Point out RS containing foods - Discuss a suggest minimum number of servings of different RS containing foods per day - Explain how these menus fit with GDM diet - Remind that increased flatulence is an expected side effect of a high fibre diet but will improve | - Participant identifies from the menus which fit best with their food preferences - Participant identifies some recipes from cookbook that they would consider making | - *High RS Sample Menus* - *Gut Feeling* cookbook | - Participant can articulate 2 or more example breakfasts, lunches, dinners & snacks that incorporate RS containing foods - Participant agrees that they can incorporate high RS foods into their diet multiple times each day |
| 10 mins | If applicable, how and when to use RS supplement. | - Explain that RS supplement is flour made from a specially bred maize - Caution to only mix into cool fluids - Encourage additional water intake to prevent constipation - Remind of possible GI symptoms, their temporary nature and to drink water | - Discuss suggestion for use of RS supplement | - *Tips for Using RS Supplement* handout | - Participant can suggest three ways they could consume the RS Starch supplement |
| 2 mins | Summary and Conclusion | - Encouragement and reassurance that they can carry out dietary interventions | - Participants asks questions if desired | - nil | - Appropriate questions asked |
| 2  mins | Next steps of the study | - Revise steps to take over next 3 days and samples to collect - Confirm appointments for Day 3 and Day 10 |  | - *Participant timetable* | - Client agrees to follow up appointment times |
