# Supplementary material for: Does a high dietary intake of resistant starch affect glycaemic control and alter the gut microbiome in women with gestational diabetes? A randomised control trial protocol
Source: BMC Pregnancy Childbirth. 2022 Jan 18;22:46. doi: 10.1186/s12884-021-04366-4 (PMC8764780; doi:10.1186/s12884-021-04366-4)

Supplement 4

MENU 1

Breakfast: 1/4 cup rolled oats* or muesli*

1 cup milk or yoghurt

1 banana*

Snack: 4 grainy crackers*

3 tablespoons hummus*

Lunch: 1 cup leftover pasta* or potato*

Small can tuna or leftover meat/chicken or 1-2 eggs

Corn on cob* or ½ cup corn kernels*

Salad with 1 tablespoon of salad dressing

Snack: Banana* and 150g flavoured yoghurt

Dinner: Meat and vegetable curry (~ 1 ½ cups)

including ½ cup chickpeas*, lentils* or beans*

2/3 cup cooked rice* or barley*

Snack: 1 cup milk and 1 grainy toast* with peanut butter*

MENU 2

Breakfast: 2 slices wholegrain bread*

120g baked beans*

1 egg

Spinach and mushrooms

Snack: ¼ cup peanuts* and banana*

Lunch: 1 cup lentils*/chickpeas*/beans*

Small serve chicken/meat/fish

Salad or vegetables

2 tablespoons hummus* as a salad dressing

2 teaspoons olive oil

½ -1 serve of fruit

Snack: Grainy or oat-based muesli bar*

Milk or milk-based coffee/Milo, etc

Dinner: 120g meat/chicken/fish

1 cup baked potato wedges*

1 medium corn on cob*

Green and orange vegetables

2 teaspoons olive oil or butter on vegetables

Snack: Banana* and yoghurt

MENU 3

Breakfast: 2 Weet-bix or ½ cup flaky cereal*

1 cup reduced fat milk

Small banana*

Snack: yoghurt

Lunch: 2 slices Burgen rye bread*

Lean meat/chicken/fish/egg

Salad

Fruit

Snack: 2 Ryvita with margarine and Vegemite

Banana

Dinner: 3 regular taco shells*

200 g mince with 3/4 cup kidney beans*

½ cup grated cheese

salad

Snack: Burgen rye bread* with peanut butter*

* Foods containing RS
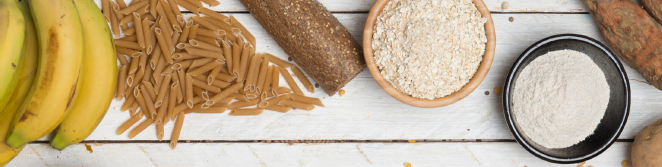

Supplement: Supplementary file 4 — Additional file 4. [file 12884_2021_4366_MOESM4_ESM.docx]
