# Supplementary material for: Does a high dietary intake of resistant starch affect glycaemic control and alter the gut microbiome in women with gestational diabetes? A randomised control trial protocol
Source: BMC Pregnancy Childbirth. 2022 Jan 18;22:46. doi: 10.1186/s12884-021-04366-4 (PMC8764780; doi:10.1186/s12884-021-04366-4)
Supplement: Supplementary file 5 — Additional file 5. [file 12884_2021_4366_MOESM5_ESM.docx]

Supplement 5

High Resistant Starch Foods

Consider adding some of these to your shopping list this week

- Rolled oats
- Raw muesli
- Firm bananas
- Potatoes
- Peas, parsnip, pumpkin
- Lentils
- Chickpeas
- Baked beans
- Other legumes
- Hummus
- Cashews, peanuts
- Rice
- Pasta, legume pasta
- Grainy bread
- Pumpernickel bread
- Grain or oat muesli bars

High RS Shopping List Version 1 28-7-2020
