# Supplementary material for: Does a high dietary intake of resistant starch affect glycaemic control and alter the gut microbiome in women with gestational diabetes? A randomised control trial protocol
Source: BMC Pregnancy Childbirth. 2022 Jan 18;22:46. doi: 10.1186/s12884-021-04366-4 (PMC8764780; doi:10.1186/s12884-021-04366-4)
Supplement: Supplementary file 6 — Additional file 6. [file 12884_2021_4366_MOESM6_ESM.docx]

**Instructions for completing a weighed food record**

Supplement 6

**Food and Glucose Record**

1. Try not to change your eating habits. This is difficult because the measuring becomes intrusive and you end up eating items that you know you can measure with ease. If you are distracted by measuring, it is better to consume your normal choices and weigh a similar-weighted food item later.

2. Record ALL that you eat and drink: a bite of somebody’s apple; a sip of soft drink; a nibble of a toddler’s leftovers; etc.

3. Only record the amount of food that you ate. If you did not consume all that you served, please subtract the weight of the leftover food on your plate to work out how much you actually ate.

4. Record your food intake as soon as possible after eating. The longer you leave it the less likely you are to be accurate.

5. If it is a commercial food item, write down the brand name and keep any nutrition information, which is on the label.

6. Weigh and/or measure food accurately. Use digital kitchen scales, metric measuring cups and spoons, or a measuring jug for liquids.

7. Record as neatly as possible so that unnecessary errors do not occur.

8. If your food was prepared by someone else, try to obtain a list of the ingredients and estimate the proportion of the whole recipe that you ate.

9. Use a new page for each day of recording.

**Remember that the Research Food Diary app might be easier as you have it with you all the time. You can take pictures of foods that you find hard to explain or log in the app and you can write notes. You could also write any extra notes or foods on these pages if you are having difficulty working out how to log them in the app.**

**Please test and record your blood glucose levels at the times indicated on this sheet.**

|  | **Time** | **List the foods/drink you consumed.** Include brand names if possible. | **How much you consumed** in grams, cups or spoons. |
| --- | --- | --- | --- |
| **Blood Glucose Level**  **Before Breakfast = ______** |  |  |  |
| **Breakfast** |  |  |  |
| **Blood Glucose Level**  **2 hrs after Breakfast = ____** |  |  |  |
| **Morning Snacks** |  |  |  |
| **Lunch** |  |  |  |
| **Blood Glucose Level**  **2 hours after Lunch = _____** |  |  |  |
| **Afternoon Snacks** |  |  |  |
| **Dinner** |  |  |  |
| **Blood Glucose Level**  **2 hours after Dinner = ____** |  |  |  |
| **Before Bed Snack** |  |  |  |
| **Extra foods or fluids** |  |  |  |

**Food and Glucose Record**

**Day:**  ___________ **Date:** __________ **Participant ID:** ________
