# Supplementary material for: Does a high dietary intake of resistant starch affect glycaemic control and alter the gut microbiome in women with gestational diabetes? A randomised control trial protocol
Source: BMC Pregnancy Childbirth. 2022 Jan 18;22:46. doi: 10.1186/s12884-021-04366-4 (PMC8764780; doi:10.1186/s12884-021-04366-4)
Supplement: Supplementary file 7 — Additional file 7. [file 12884_2021_4366_MOESM7_ESM.docx]

Supplement 7

Adding Maize Starch to Food

The starch should only be added to cool or cold foods.

Try stirring the starch into 2-3 tablespoons of a liquid or semi-liquid food. Sometimes a dash of water or milk will help to return the food to its usual consistency.

If adding to a large volume of liquid, stir into a few tablespoons of that liquid first, then top up with the remainder.

Starch can be added to foods like these. You might come up with a few more.

- Mashed banana
- Yoghurt – plain or flavoured
- Mixed with milk or yoghurt then added to cereal
- Milk or soy milk
- Flavoured milk drinks e.g. cold Milo
- Smoothies
- Cool custard
- 150g cool rice pudding
- 120g puree apple, pear or other fruits
- 2 tablespoons Hummus plus 1 tablespoon water

Remember to drink lots of water when you are consuming extra resistant starch.

**Remember to drink lots of water when you are consuming extra resistant starch**
