# Supplementary material for: Does a high dietary intake of resistant starch affect glycaemic control and alter the gut microbiome in women with gestational diabetes? A randomised control trial protocol
Source: BMC Pregnancy Childbirth. 2022 Jan 18;22:46. doi: 10.1186/s12884-021-04366-4 (PMC8764780; doi:10.1186/s12884-021-04366-4)
Supplement: Supplementary file 8 — Additional file 8. [file 12884_2021_4366_MOESM8_ESM.docx]

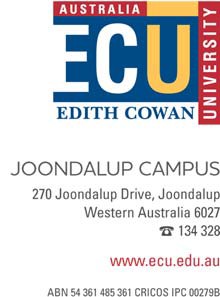
Supplement 8

##

## Stool Collection and Storage Information

**Why do we want a stool sample?**

We know this is an unusual request! But in your bowel there are many bacteria that play an important role in your overall health. We can use stool samples to assess the amount and type of bacteria in your gut.

**What we need:**

We would like you to collect your first stool sample of the day. If you do not have a bowel action during that day, please continue until you have a bowel action.

**Please place ice blocks in freezer 24 hours prior to collection**

**What we don’t need:**

For analysis, it is important **not** to contaminate the stool sample with urine. If it does happen, please still collect the sample and we will determine if the sample can be analysed. Please do not allow toilet paper to touch the sample as this can affect the analysis.

**What is in your stool kit?**

Your stool sample collection pack will include the following items: one pre‐labelled blue collecting bag to go under the toilet seat one cable tie to seal the bag, one large individual zip lock bag so that the sample is sealed into several bags.

**How to collect the stool sample:**

1. Please write the **date, time** and your **participant ID number** clearly on the label on the bag.

***GDM Resistant Starch Study***

***ID:***

***Date: Time:***

1. Please empty your bladder into the toilet before collecting samples (urine will contaminate the sample).
2. Use the blue plastic bag to line the toilet for collection – as shown in the photo. Please keep the bag above the water in the pan. To avoid urine contamination, you may prefer to line only the back half of the toilet with the blue plastic bag to allow space at the front for urine.


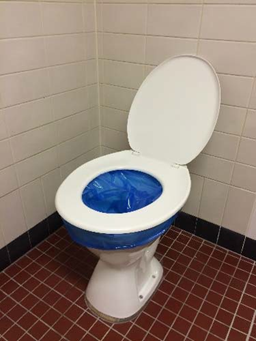
For women: You may need to use a pad or toilet paper or a paper cup to absorb or collect any urine during your bowel action.

1. **Do not allow any toilet paper** to be collected with the sample.
2. **Do not cut the top off the bag** as the laboratory weighs the sample and the bag.
3. Please check that the label is visible and not caught in the tie. Expel as much air as possible before twisting the bag and folding over. Securely tie the blue bag just above the sample with a cable tie. Please ensure that the cable tie is locked tight.
4. Place one blue bag inside of one clear plastic snap-lock bag and seal. **Do not use any other bags other than those provided.**
5. Immediately place the sample into the cooler bag with the ice packs.
6. Please return the cooler bag to us as soon as possible as the samples will only be kept cool for 24 hours in the cooler bag. This means the stool sample will be kept cold and provide the best results for your gut health test.

If you have any questions at any time, please contact the following researchers:
