# Supplementary material for: Does a high dietary intake of resistant starch affect glycaemic control and alter the gut microbiome in women with gestational diabetes? A randomised control trial protocol
Source: BMC Pregnancy Childbirth. 2022 Jan 18;22:46. doi: 10.1186/s12884-021-04366-4 (PMC8764780; doi:10.1186/s12884-021-04366-4)
Supplement: Supplementary file 9 — Additional file 9. [file 12884_2021_4366_MOESM9_ESM.docx]

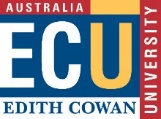
Supplement 9

**Infant Stool Sample Collection Procedure**


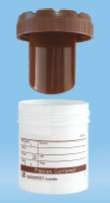
**To collect this sample, we have supplied you with the following:**

- A liner for a nappy
- Two plastic sample containers each with a brown screw-cap and an attached spoon for scooping
- Disposable gloves
- A small transparent plastic bag for transport
- Cooler bag
- Two ice packs

**Please follow the instructions below** to collect two stool samples from a nappy (1 stool sample per brown screw-cap container).

**Aim to collect the sample** within 24 hours before your appointment (i.e., either the day before or the day of your appointment).

**Within 24 hours of your appointment**

Place the nappy liner on the inside of a clean nappy. Place the lined nappy on your child.

**Steps for Stool Sample collection**

1. Wash your hands and put on disposable gloves prior to collecting the stool sample.
2. Use a stool sample from a clean nappy liner, preferably free from urine.
3. Unscrew the top of one brown screw-cap container.
4. Using the spoon inside the brown lid, completely fill the spoon with stool. **IMPORTANT: Please make sure you scoop all the way through the sample (i.e., cross-sectionally) rather than just scraping across the surface of the sample.**
5. Insert the filled spatula into the sample container and close the brown screw-cap lid.
6. Using the second brown-cap container to collect another sample of stool by repeating steps 4-6.
7. Place the two filled containers into the plastic bag.
8. Immediately place the bag of samples into the cooler bag with the ice packs.
9. Dispose of the nappy and gloves in your household bin - do not flush. Wash your hands.
10. Record the date, time and type of stool sample collection on the *Stool Sample Collection Record* attached, indicating on the Bristol Stool Form Scale (for children) which type of stool most closely describes the stool just passed by your child.
11. Please bring the cooler bag containing stool samples and the *Stool Sample Collection Record* to your appointment.
