# Supplementary material for: Does a high dietary intake of resistant starch affect glycaemic control and alter the gut microbiome in women with gestational diabetes? A randomised control trial protocol
Source: BMC Pregnancy Childbirth. 2022 Jan 18;22:46. doi: 10.1186/s12884-021-04366-4 (PMC8764780; doi:10.1186/s12884-021-04366-4)
Supplement: Supplementary file 10 — Additional file 10. [file 12884_2021_4366_MOESM10_ESM.docx]

Supplement 10


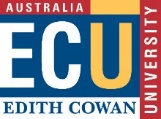
**Breastmilk Sample Collection Procedure**

Thank for your participation in this study. Please follow the instructions below to collect 10 mL (2 filled collection tubes) of breastmilk after feeding your baby. We will be using the breastmilk collected to obtain a representative bacterial profile of breastmilk, and to determine if that influences the bacterial profile of your child’s gut. Breastmilk is easily contaminated by bacteria from the environment, so please adhere to the instructions so that we are able to minimise detection of bacteria not originating from breastmilk.

**To collect this sample, we have supplied you with the following:**

- Sterile disposable gloves x 1 pair
- 5 ml collection tubes x 2
- Zip-lock plastic bag x 1
- Cooler bag with 2 ice packs (Please place ice packs into your freezer when you get home)

**Please follow the instructions below** to collect two breastmilk samples in the morning, after feeding your baby, on the same breast.

**Within 48 hours of your appointment.**

**Steps for Breastmilk Sample collection**

1. CLEAN HANDS: **After feeding** your baby in the **morning**, **wash hands** thoroughly with soap for 20 seconds. Dry hands with clean paper towels after washing. Put on gloves.
2. EXPRESS AND COLLECT MILK **AFTER** FEEDING (10 mL): Collect milk by hand expression or using a sterilised pump into the sterile collection tubes. Please use the **same breast** for collection. **Be careful not to touch the inside of the collection tube** when collecting milk. Collect 5 mL of breastmilk per tube (fill the entire collection tube).
3. FREEZE AS SOON AS POSSIBLE: Place the 2 breastmilk collection tubes immediately into the zip-lock plastic bag and then into the freezer. For transportation to the hospital, place the plastic bag with breastmilk samples into the cooler bag containing frozen ice packs.
4. FILL IN THE BREASTMILK COLLECTION RECORD: Fill in the *Breastmilk Collection Record* attached. Pass the cooler bag with the 10 mL of collected breastmilk and the *Breastmilk Collection Record* to researchers during your appointment.
