# Supplementary material for: Does a high dietary intake of resistant starch affect glycaemic control and alter the gut microbiome in women with gestational diabetes? A randomised control trial protocol
Source: BMC Pregnancy Childbirth. 2022 Jan 18;22:46. doi: 10.1186/s12884-021-04366-4 (PMC8764780; doi:10.1186/s12884-021-04366-4)
Supplement: Supplementary file 11 — Additional file 11. [file 12884_2021_4366_MOESM11_ESM.docx]

Supplement 11

**ID:**

| **Bowel Symptoms Questionnaire**  Thank you for taking the time to complete this questionnaire.  All personal details contained in this questionnaire will be treated strictly confidential. All the questions asked are designed to aid our research.  You may find some of the details required are hard to recall – if so, please answer these to the best of your ability by making estimates that are as accurate as possible. It is to be expected that your symptoms might vary over time, so try and answer the questions based on how you currently feel (i.e. over the last 10 days or so).  **Instructions**   1. For questions where a number of different responses are a possibility, please circle the response most appropriate to you. 2. Some questions will require you to write in an response. 3. Some questions require you to put a cross on a line, which enables us to judge the severity of a particular problem.   *For example:* How severe is your abdominal (tummy) pain?  Please place your cross (🗶) anywhere on the line between 0-100%, in order to indicate as accurately as possible the severity of your symptom.  This example shows a severity of approximately 90%  0% I-----------------------------------------------------------------------I 100%  No pain not very quite severe very  severe severe severe  **Question 1.**  (a) Have you suffered from abdominal (tummy) pain in the past week?  Please circle **Yes** Please complete parts (b) & (c).  **No** Please go to Question 2.  (b) How severe is your abdominal (tummy) pain?  0% I-----------------------------------------------------------------------I 100%  No pain not very quite severe very  severe severe severe  (c) How many days out of the last 10 days did you get tummy pain?  **Question 2.**   1. Apart from being pregnant, do you currently suffer from abdominal distention? (bloating or swollen tummy)   Please circle **Yes** Please complete section (b)  **No** Please go to Question 3.   1. How severe is your abdominal distention/bloating?   0% I-----------------------------------------------------------------------I 100%  No pain not very quite severe very  severe severe severe  **Question 3.**  How satisfied are you currently with your bowel habits?  0% I-----------------------------------------------------------------------I 100%  Very happy quite unhappy very  happy unhappy  **Question 4.**  How much have your bowel habits been affecting your life in general?  0% I-----------------------------------------------------------------------I 100%  Not at all not much quite a lot completely  **Thank you**  Adapted from: Francis, C. Y., Morris, J., & Whorwell, P. J. (1997). The irritable bowel severity scoring system: a simple method of monitoring irritable bowel syndrome and its progress. *Alimentary Pharmacology & Therapeutics, 11*(2), 395-402. doi:10.1046/j.1365-2036.1997.142318000.x |
| --- |
