# Supplementary material for: Does a high dietary intake of resistant starch affect glycaemic control and alter the gut microbiome in women with gestational diabetes? A randomised control trial protocol
Source: BMC Pregnancy Childbirth. 2022 Jan 18;22:46. doi: 10.1186/s12884-021-04366-4 (PMC8764780; doi:10.1186/s12884-021-04366-4)
Supplement: Supplementary file 12 — Additional file 12. [file 12884_2021_4366_MOESM12_ESM.docx]

Supplement 12

**Bowel Symptoms, Medication and Exercise Record**

**Day** ____________  **Date** ___________ **ID** ___________

| **Bowel Movements** | 1 | 2 | 3 |
| --- | --- | --- | --- |
| **Bristol Stool Chart Score**  (Score each motion using **Bristol Stool Chart** and write score of 1-7 in box) |  |  |  |
| **Ease of Stool Passage**  (Score each motion using the scale below and write a score of 1-5 in the box) |  |  |  |

**Ease of Stool Passage Score:** 1 = Very easy 2 = Easy 3 = Neither easy nor difficult 4 = Difficult 5 = Very difficult

| **Bowel Symptoms** (compared to usual) | **Flatulence** | **Abdominal rumbling** | **Abdominal cramping** | **Abdominal bloating** | **Nausea** | **Diarrhoea** | **Constipation** |
| --- | --- | --- | --- | --- | --- | --- | --- |
| 0 = No change  1 = Mild  2 = Moderate  3 = Severe | 0 1 2 3 | 0 1 2 3 | 0 1 2 3 | 0 1 2 3 | 0 1 2 3 | 0 1 2 3 | 0 1 2 3 |

| **Medications taken today:** | **Resistant Starch supplements consumed today (if applicable):** |
| --- | --- |
|  | **€** Scoop 1 (tick if consumed)  **€** Scoop 2 |
| **Exercise performed today:** | **Other comments:** |
| Type -  Time -  Duration - |  |
