# Supplementary material for: Does a high dietary intake of resistant starch affect glycaemic control and alter the gut microbiome in women with gestational diabetes? A randomised control trial protocol
Source: BMC Pregnancy Childbirth. 2022 Jan 18;22:46. doi: 10.1186/s12884-021-04366-4 (PMC8764780; doi:10.1186/s12884-021-04366-4)
Supplement: Supplementary file 14 — Additional file 14. [file 12884_2021_4366_MOESM14_ESM.docx]

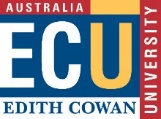
Supplement 14

**
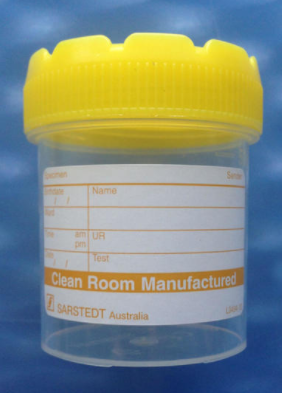
Urine Sample Collection Procedure**

**To collect this sample, we have supplied you with the following:**

- One specimen pot
- A small transparent plastic bag for transport
- Cooler bag
- Two ice packs

**Please follow the instructions below and collect a sample of your first urine void for the day** within 24 hours before your appointment (i.e., either the day before or the day of your appointment).

**Within 24 hours of your appointment**

**Steps for Urine Sample collection**

1. Wash your hands.
2. Open the specimen pot. Do not touch the inside of the pot or the inside of the lid.
3. Place the pot into your stream of urine to collect a sample. Try to fill at least half of the pot.
4. Firmly screw the lid back onto the pot.
5. Use toilet paper to wipe any spilled urine off the closed pot and place it into the plastic bag.
6. Immediately place the bag containing the sample into the cooler bag with the ice packs.
7. Record the date and time of the urine sample collection on the *Urine Sample Collection Record* attached.
8. Please bring the cooler bag containing urine sample and the *Urine Sample Collection Record* to your appointment.

**Urine Sample Collection Record**

Participant ID

1. Date of urine sample collection: __ __ / __ __ / __ __
2. Time of urine sample collection: ___ ___ : ___ ___ AM / PM
