# Supplementary material for: Does a high dietary intake of resistant starch affect glycaemic control and alter the gut microbiome in women with gestational diabetes? A randomised control trial protocol
Source: BMC Pregnancy Childbirth. 2022 Jan 18;22:46. doi: 10.1186/s12884-021-04366-4 (PMC8764780; doi:10.1186/s12884-021-04366-4)
Supplement: Supplementary file 15 — Additional file 15. [file 12884_2021_4366_MOESM15_ESM.docx]

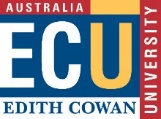
Supplement 15

**Infant Urine Sample Collection Procedure**

**
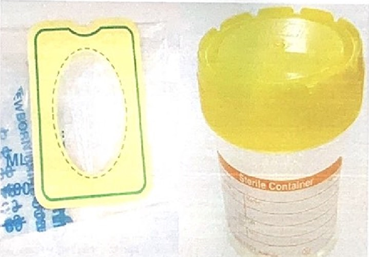
To collect this sample, we have supplied you with the following:**

- Urine collection bag x 1
- Plastic sample jar x 1
- Small transparent plastic bag for transport x 1
- Cooler bag x 1
- Ice packs x 2

**Please follow the instructions below** to collect one urine sample within 24 hours of your appointment (i.e., either the day before or the day of your appointment).

**Within 24 hours of your appointment**

**Steps for Infant Urine Sample collection**

Please do not touch inside the bag, jar or lid.

1. Wash your hands with soap and dry well.
2. Remove urine bag from package and unfold it.
3. Clean and dry baby’s genital area.
4. Remove yellow sticker from urine bag.
5. Apply urine bag to baby’s genital area making sure there is a seal.
6. Apply nappy as usual.
7. Once a urine sample is obtained:
   1. Gently remove the urine bag from your baby.
   2. Pour the urine into the specimen jar.
   3. Screw on the lid firmly, place the jar in the plastic bag and then into the fridge.
8. Record the date and time of urine sample collection on the *Infant Urine Sample Collection Record* attached
9. Please bring the cooler bag containing urine sample and the *Infant* *Urine Sample Collection Record* to your appointment.

**Infant Urine Sample Collection Record**

Participant ID

1. Date of urine sample collection: __ __ / __ __ / __ __
2. Time of urine sample collection: ___ ___ : ___ ___ AM / PM
