# Supplementary material for: Does a high dietary intake of resistant starch affect glycaemic control and alter the gut microbiome in women with gestational diabetes? A randomised control trial protocol
Source: BMC Pregnancy Childbirth. 2022 Jan 18;22:46. doi: 10.1186/s12884-021-04366-4 (PMC8764780; doi:10.1186/s12884-021-04366-4)
Supplement: Supplementary file 16 — Additional file 16. [file 12884_2021_4366_MOESM16_ESM.docx]

Supplement 16

World Health Organization Trial Registration Data Set Summary

| Data Category | Information |
| --- | --- |
| Primary Registry and Trial Identifying Number | Australian New Zealand Clinical Trial Registry ACTRN12620000968976p |
| Date of Registration in Primary Registry | 28 September, 2020 |
| Secondary Identifying Numbers | WHO Universal Trial Number U1111-1254-8361 |
| Source(s) of Monetary or Material Support | Australian Government Research Training Program (RTP) Scholarship  Chief Allied Health Officer, Western Australian Department of Health  Edith Cowan University, Western Australia  South Metropolitan Health Service, Western Australia |
| Primary Sponsor | Edith Cowan University, Western Australia |
| Secondary Sponsor(s) | South Metropolitan Health Service, Western Australia |
| Contact for Public Queries | Cathy Latino, Department of Dietetics, Fiona Stanley Hospital, 11 Robin Warren Drive, Murdoch, 6150, Western Australia.  Telephone: +61 8615 2222 Email: Cathy.Latino@health.wa.gov.au |
| Contact for Scientific Queries | Cathy Latino, Department of Dietetics, Fiona Stanley Hospital, 11 Robin Warren Drive, Murdoch, 6150, Western Australia.  Telephone: +61 8615 2222 Email: Cathy.Latino@health.wa.gov.au |
| Public Title | The Effect of a High Dietary Intake of Resistant Starch on Blood Glucose Levels in Women with Gestational Diabetes |
| Scientific Title | The Effect of Dietary Resistant Starch on Maternal Glycaemia and Gut Microbiome in Gestational Diabetes |
| Countries of Recruitment | Australia |
| Health Condition(s) or Problem(s) Studied | Gestational Diabetes Mellitus (GDM) |
| Intervention(s) | Intervention group 1: Diet high in resistant starch-rich foods  Intervention group 2: Diet high in resistant starch-rich foods supplemented with 40 g high-amylose maize starch providing 16 g resistant starch  Control: Standard dietary management of GDM |
| Key Inclusion and Exclusion Criteria | Inclusion: Women newly diagnosed GDM, diagnosed between 24-30 weeks gestation, ≥ 18 years.  Exclusion: Recent antibiotic use, significant mental or physical health condition, bariatric surgery, gastrointestinal condition, laxative or probiotic use |
| Study Type | Open-label, parallel-group design  Allocation: Block randomisation by BMI category  Masked: No  Purpose: Improve glycaemic control |
| Date of First Enrolment | May 2021 |
| Target Sample Size | 90 |
| Recruitment Status | Recruiting |
| Primary Outcome(s) | Fasting blood glucose at Day 10 |
| Key Secondary Outcomes | Fasting blood glucose at Day 56  Post-prandial blood glucose levels at Day 10  Rate of requirement for glucose lowering medication at Day 10  Maternal gut microbiota at Day 10 |
| Ethics Review | Protocol Version 2.3 approved by South Metropolitan Health Service Human Research Ethics Committee (RGS0000004140), 14 Barry Marshall Parade, Murdoch, Western Australia, 6150 on 27 April 2021.  Approved by Edith Cowan University Human Research Ethics Committee (2020-01960-LATINO), 270 Joondalup Drive, Joondalup, Western Australia, 6027, on 7 May 2021 |
| Completion date | Trial not completed |
| Summary Results | Trial not completed |
| IPD sharing statement | Plan to share IPD: No |
